# Valorization of *Eichhornia crassipes* for the production of cellulose nanocrystals further investigation of plethoric biobased resource

**DOI:** 10.1038/s41598-024-62159-z

**Published:** 2024-05-29

**Authors:** Mohamed H. Hemida, Hesham Moustafa, Sherif Mehanny, Mohamed Morsy, Eid N. Abd EL Rahman, Mohamed M. Ibrahim

**Affiliations:** 1 Agricultural Engineering Department, Faculty of Agriculture, Cairo University, Giza, Egypt; 2 Department of Polymer Metrology & Technology, National Institute of Standards (NIS), Tersa Street, El Haram, P.O Box 136, Giza, 12211 Egypt; 3 Bioanalysis Laboratory, National Institute of Standards (NIS), Tersa Street, El Haram, P.O Box 136, Giza, 12211 Egypt; 4 Department of Mechanical Design and Production, Faculty of Engineering, Cairo University, Giza, Egypt; 5 Building Physics and Environment Institute, Housing and Building National Research Center (HBRC), Dokki, Giza, Egypt; 6 Nanotechnology Research Center (NTRC), The British University in Egypt (BUE), El Sherouk City, Suez Desert Road, Cairo, 11837 Egypt

**Keywords:** Agro-residues, *Eichhornia crassipes (Nile roses)*, Cellulose nanocrystals extraction, Raman spectroscopy, Thermogravimetric analysis, Engineering, Nanoscience and technology

## Abstract

Chemical processing is among the significant keys to tackle agro-residues utilization field, aiming to obtain value-added materials. Extraction of cellulose nanocrystals (CNCs) is an emerging route to valorize lignocellulosic wastes into high value particles. In this investigation, effect of acidic hydrolysis duration was monitored on size and morphology of obtained crystals; namely: CNCs from Nile roses fibers (NRFs) (*Eichhornia crassipes)*. Different acidic hydrolysis duration range or different characterization techniques set this article apart from relevant literature, including our group research articles. The grinded NRFs were firstly subjected to alkaline and bleaching pretreatments, then acid hydrolysis process was carried out with varied durations ranging from 5 to 30 min. Microcrystalline cellulose (MCC) was used as reference for comparison with NRFs based samples. The extracted CNCs samples were investigated using various techniques such as scanning electron microscopy (SEM), Atomic force microscopy (AFM), Raman spectroscopy, and thermogravimetric (TGA) analysis. The figures gotten from SEM and AFM depicted that NRFs based CNCs appeared as fibril-like shapes, with reduced average size when the NRFs underwent pulping and bleaching processes. This was indicated that the elimination of hemicellulose and lignin components got achieved successfully. This outcome was proven by chemical composition measurements and TGA/DTG curves. On the other hand, AFM-3D images indicated that CNCs topology and surface roughness were mostly affected by increasing hydrolysis durations, besides smooth and homogeneous surfaces were noticed. Moreover, Raman spectra demonstrated that the particle size and crystallinity degree of NRFs based CNCs can be affected by acidic hydrolysis durations and optimum extraction time was found to be 10 min. Thermal stability of extracted CNCs-NRFs and CNCs-MCC was measured by TGA/DTG and the kinetic models were suggested to identify the kinetic parameters of the thermal decomposition of CNCs for each acid hydrolysis duration. Increasing hydrolysis duration promoted thermal stability, particularly for NRFs based CNCs. Results showcased in this article add new perspective to Nile rose nanocellulose and pave down the way to fabricate NRFs based humidity nano-sensors.

## Introduction

Environmental friendliness has grown tremendously as an impactful factor in engineering solutions and material selection. Holding environmental conferences (e.g., COP27) that tailor agreements and recommendations, which will consequently be enforced on governments and private sectors. Consequently, nanocellulose-based agro-waste has attracted much interest in a variety of disciplines due to its unique properties comparable with classical petroleum-based materials^1,2^. In addition to its renewability, aboundingly, nontoxicity, and biodegradability. Many novel series of biodegradable materials that can be lastly decomposed to carbon dioxide, water, and humus have developed. These advantages could open opportunities for their utilize in a broad spectrum of applications including food packaging, smart farming and industrial composting. Nanocellulose is considered as lignocellulosic material genre as it is the most plethoric biomaterial worldwide. More than 1.3 × 10^10^ metric ton per annum is the global production of lignocellulose^3,4^. It is the fundamental constituent of terrestrial plants and aquatic algae in all farming, forest, as well as marine ecosystems. Lignocellulose encompasses three components; namely: cellulose, hemicellulose, and lignin. Cellulose counts for the mechanical stiffness of lignocellulosic texture, it is a naturally polymerized chain of glucose monomers. Also it provides plant cell walls with mechanical strength and other properties including cell expansion, water movement and defense responses against pests or microbes^5^. Hemicellulose is a heterogeneous polymeric substance consisting of C5-C6 monomers, it surrounds cellulose fibrils and fibers^6^. Lignin is an aromatic compound of high molecular weight (100 KDa macromolecules), which winds cellulose and hemicellulose, shielding them due to its hydrophobic and thermo-resistant behavior^7^. In this article, we will emphasize more on cellulose, as we destruct it down to nanoscale. Nile roses (*Eichhornia Crassipes*), the plant which grows widely on surfaces of fresh water, wastes more than 3.5 billion m^3^ of Egyptian fresh water per annum, in addition to blocking navigation in Nile stream and darkening deep water inhibiting photosynthesis^8^. Which necessitates extensive removal of this parasitic plant. Due to some water contaminants accumulation in the Nile rose, it cannot be used as animal feed. Hence, finding other routes to exploit this mega size biomass becomes more obtrusive. Nile roses prove high reliability in biocomposite fabrication and hopefully nanocellulose mining^9,10^.

Nanocellulose word is becoming more buzzy during last decade due to challenging properties, availability, which led to unbeatable versatility^11^. Ultra-high tensile strength (exceeding 9 GPa), having diverse particle size, crystallinity content, chiral nematic properties and minimal toxicity (natural source), all combined nominated nanocellulose as future material for diverse applications^12,13^. Nanocellulose can be prepared by one of three methods: Nanocellulose can be prepared by either one of these three methods: chemical, mechanical, and biological, or chemo-mechanical method. Chemical method is less costly and less energy intensive process, it yields high crystalline, low aspect ratio cellulose nanocrystals^14^. Mechanical is higher in cost and energy, as it requires more sophisticated mechanical equipment (disc milling, microfluidic channels, homogenizer, and ultrasonicator), mechanical extraction yields less crystalline high aspect ratio nanofibril. Bacterial method is simply assimilating glucose monomer within unicellular organism and excreting linear chains of ultrapure nanocellulose chains called bacterial nanocellulose (BNC)^15,16^. BNC conveys zero toxicity and perfect biocompatibility, which promotes its biomedical applicability^17^. Applications of nanocellulose encompass plastic reinforcement, chemical modification, transparent plastics for lenses, hygiene pads, dental implants, and wound healing^18,19^.

In this research, the Nile roses or *Eichhornia Crassipes* fibers were selected because it has high growth rate which causes disastrous repercussions for Egyptian fresh water, despite low lignin concentration, renewability and low-price as a raw material. Therefore, employing Nile rose as nanocellulose raw material provides double benefits. The main objective of this article is to investigate potential of lignin-poor Nile roses as a precursor for nanocellulose via chemical methods. Nile roses fibers will undergo chemical processes before acidic hydrolysis as what will be explained later in the materials section. In this article we attempted to conduct advanced characterization including SEM, AFM, and Raman spectroscopy, aiming to have deeper understanding to Nile roses processing into nanocellulose. The thermal degradation of the prepared CNCs specimens was also discussed. Some useful thermal parameters represented in activation energy, entropy, enthalpy, and Gibbs free energy were calculated and discussed.

## Materials and experimental techniques

### Materials

Nile roses plants were collected from the channels of the Nile water canals in Beni Suef Governorate, Egypt and dried through solar dehydration process until reaching constant weight. The dehydration period lasted one month on house roof, followed by further drying in an oven at 105 °C for 24 h. Sodium hydroxide with a dosage of 98.6% was purchased from Fisher Scientific. Acetic acid with 98% purity and sodium chlorite (NaClO_2_, 98.8%), used for the bleaching process after fiber pulping treatment, were obtained from Sigma-Aldrich, Egypt. Microcrystalline cellulose (MCC) was purchased from Avi-Chem Industries, Maharashtra, India and used for comparison with CNCs extracted from NRFs.

### Extraction process of cellulose nanocrystals from NRFs

**Figure 1:**
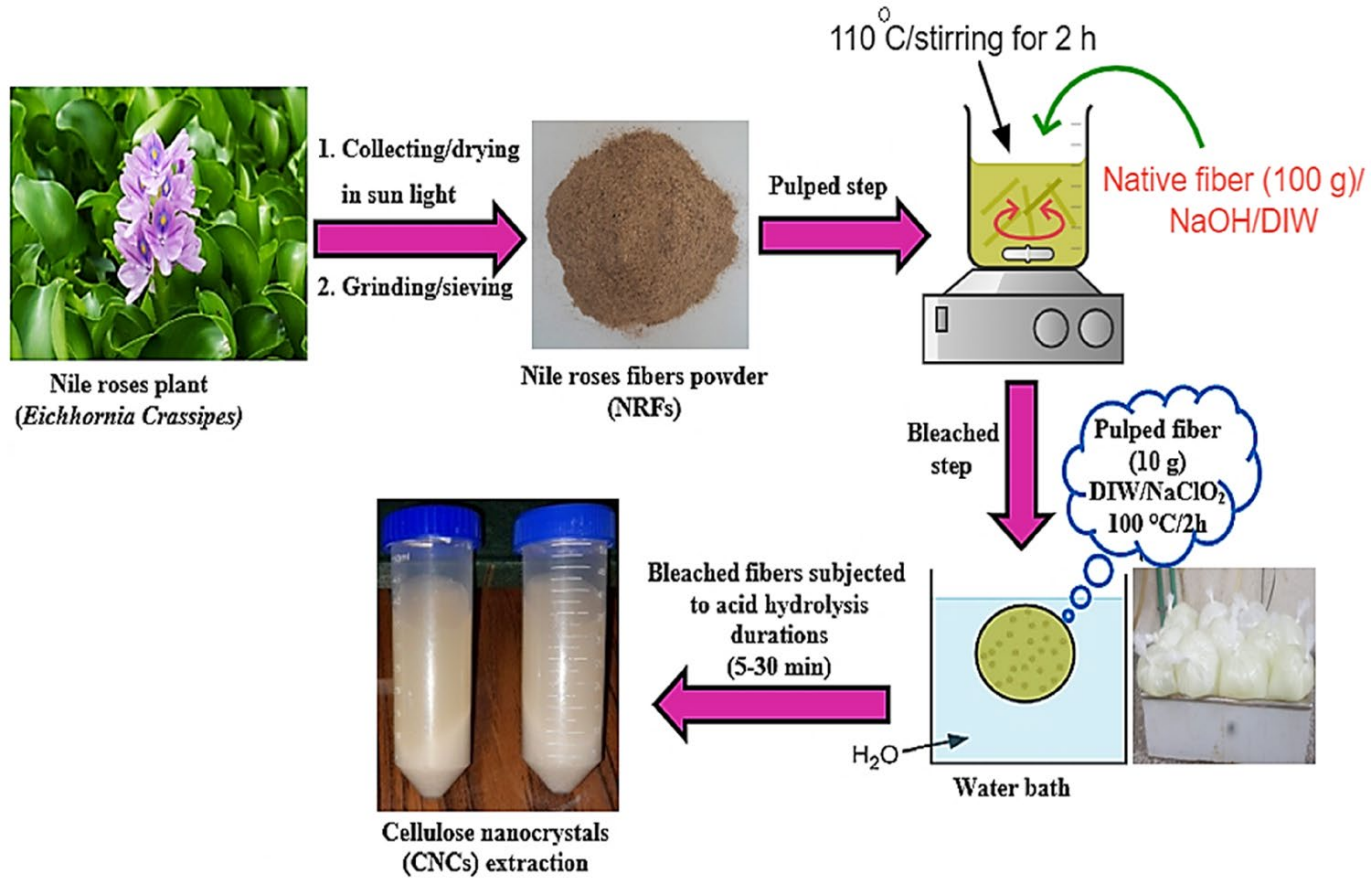
Extraction steps of cellulose nanocrystals (CNCs) from Nile roses fibers (NRFs).

After complete drying, the Nile rose leaves were cut into small pieces and then ground using a laboratory mill (see Fig. 1). The pulping process was performed by mixing 100 g of native fiber with 1.750 L of deionized water (DIW) and 20 g of NaOH. The mixture was heated up to 110 °C for 2 h, and then the fibers were washed thoroughly and dried in an electric oven to a constant weight. The pulped fibers were subjected to a bleaching treatment, performed by mixing 10 g of pulped fiber with 400 mL of DIW with 1.5 g of NaClO_2_ (sodium chlorite) and 15 mL of acetic acid. Then, they were put in a water bath for 2 h at 100 °C. The bleaching process was repeated 10 times to obtain perfect white fibers. Both bleached NRFs residues and lab microcrystalline cellulose (MCC) were subjected to acid hydrolysis by adding 3 g of (bleached NRFs or MCC) to 60 mL of 60% w/v H_2_SO_4_ and heated to 45 °C for 5, 10, 20, and 30 min under mechanical stirring. Comparably low durations were chosen to cope with NRF biomass lesser recalcitrance (compliance), as the entire bleached NRF biomass vanishes when prolonging durations after 30 min. The acid hydrolysis reaction was interrupted by quenching in 1200 mL of ice. Subsequently, the resulting suspension was centrifuged at 4500 rpm for 30 min, simultaneously adding 5% (w/v) NaHCO_3_ until the pH of the suspension reached 7–8. After that, the supernatant was decanted and replaced with DIW. A 50 mL tube was shaken and recentrifuged. The last two steps were repeated 1–2 times and then the concentrate was stored in glass vials in a laboratory fridge until further investigation was performed. In order to complete this process, many attempts were made in the preliminary phase; nevertheless, generally in documented experiments, three experimental repetitions were conducted for all specimens.

### Scanning electron microscopy (SEM)

The morphological shape, fiber composition and size of (Native-Pulped-Bleached) NR fibers were characterized using field emission scanning electron microscopy, (FE-SEM), model Quattro S, Thermo Scientific. The electron beam spot size was carefully chosen to be in the range of 3–3.5 to improve the quality of the images. All specimens were coated with a thin gold couch using Sputter-coating equipment before the observation to prevent electrostatic charge during monitoring^20,21^. During SEM observation, fibers samples were fixed on the SEM samples holder and analyzed without any spectroscopic technique. The acceleration voltage was 10 kVA. The EDX (elemental analysis) analysis unit was attached to the SEM.

### Atomic force microscopy (AFM)

The morphological structures and dimension scales of extracted CMCs samples were investigated using an AFM (AutoProbe CP-Research Probe Head) manufactured by Thermo-Microscope operated in contact mode using Silicon Nitride Probe Model MLCT, Bruker. Proscan 1.8 software was used for controlling the scan parameters (i.e., contact mode, scan area 20 × 20 μm^2^, scan rate 1 Hz, and number of data points of 256 × 256 points), whereas IP 2.1 software was used for image analysis. A 0.01% mass fraction of each specimen was dispersed in double-distilled H_2_O (ddH_2_O) and sonicated for 10 min at 60% amplitude to minimize the number of bundles and aggregates of CNCs. Then, this solution was diluted tenfold and sonicated for a few minutes. One drop from the suspension was deposited on a glass microscope slide with an approximate area of 1 cm^2^ and allowed to dry completely at normal conditions in a clean area prior to the observation. At least three images were taken for each specimen.

### Raman spectroscopy

Raman spectra were used to investigate the structural and the chemical functionalities of extracted CNCs either from Nile roses fibers or from MCC using a Confocal Raman Imaging Microscope (WITec Alpha, Model 300R, Germany), with excitation laser power of 50 mW at 532 nm and diode pumped Nd:YAG laser^22^. All specimen films were scanned in the range of 50–3600 cm^-1^. For microscope images, all specimen films were photographed with a microscope camera with high resolution to observe the structural, chemical details and spatial.

### Chemical composition analysis of NRFs

The ratios of the cellulose, hemicellulose, and lignin in the *Eichhornia Crassipes* fibers prior to and after pretreatments were estimated by the Technical Association of the Pulp and Paper Industry (TAPPI) test method^23,24^. At least duplicates for each specimen were performed and the average was registered.

### Thermal analysis (DTA and TGA)

The differential thermal analysis (DTA) and gravimetric thermal analysis (TGA) was measured from room temperature up to 800 °C with a constant heating rate of 10 °C/min under N_2_ atmosphere. The data for DTA and TGA were collected by STD-Q600 instrument^25^

## Results and discussion

### Field emission scanning electron microscopy (FE-SEM)

**Figure 2:**
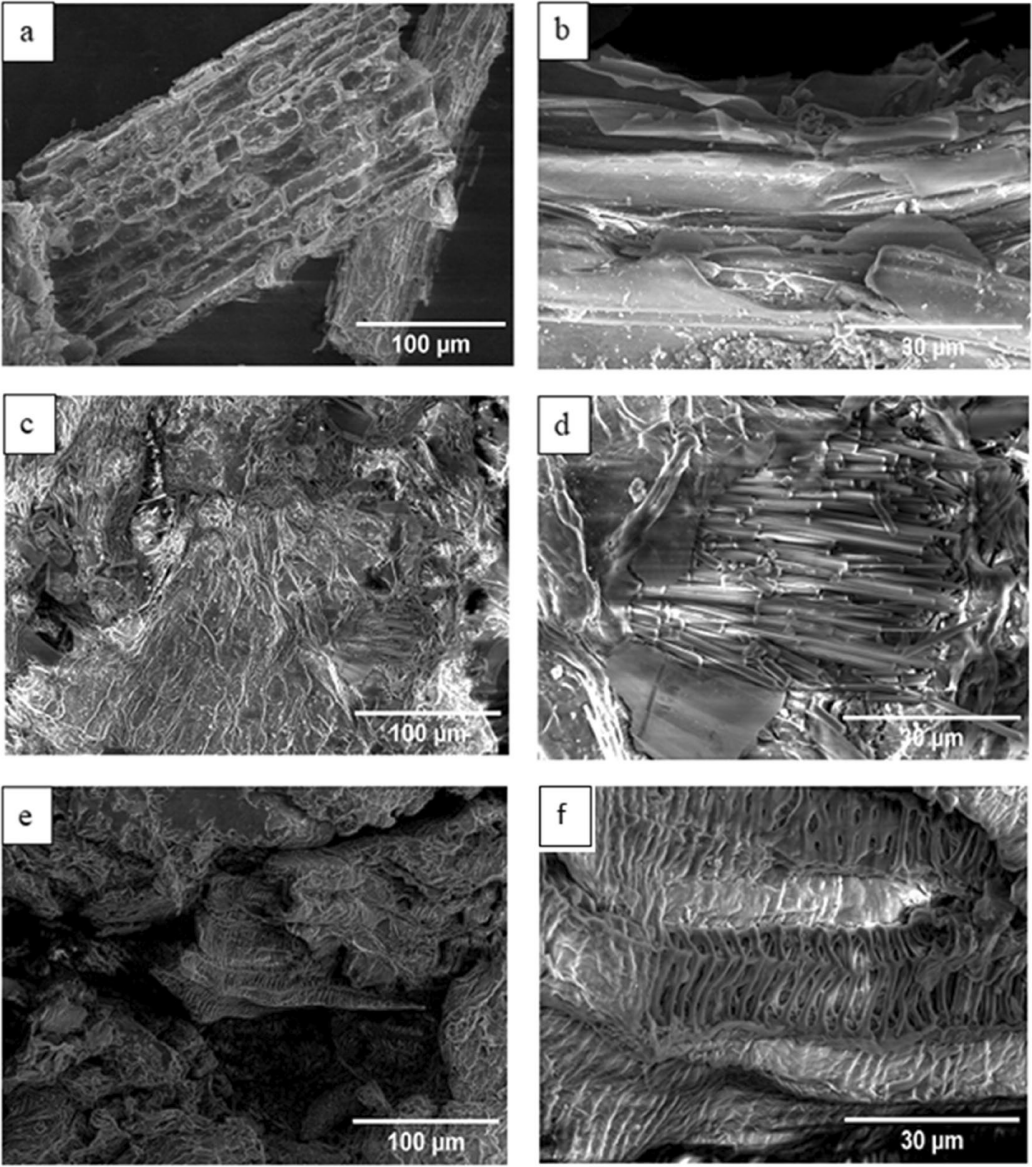
FE-SEM images of fibers: (**a**-**b**) Native fiber, (**c**-**d**) Pulped fiber, (**e**–**f**) Bleached fiber.

FE-SEM analysis shows the changes that occur in morphological shape, size and fiber composition of Nile rose plant (*Eichhornia crassipes*) under the influence of various chemical treatments (pulping and bleaching). The morphological shape and fiber composition in native fiber are shown in Fig. 2a-b. Native fiber completely appeared in solid form, and this indicates the network of cellulose, lignin, and hemicellulose that constitute native biomass. The shape of the native fiber was only conventional-like fiber (not yet fibrillated). The diameter of the fiber was measured by ImageJ program to be found 142 ± 0.2 µm. FE-SEM analysis on pulping treatment showed that the fiber breaks down severely into lower scale fibrils, after undergoing pulping, as shown in Fig. 2c-d. FE-SEM images showed that the pulped fiber contains voids, not a blind body, and presence of these voids may be interpreted by removal of lignin and hemicellulose. The average lower scale fibril diameter was 2.7 ± 0.05 µm. Figure 2e–f exhibited bleached fiber skeleton-like features and their higher count of voids than pulped fiber. Bleached fibers photos are indicators of intense removal of lignin and hemicellulose, and hence sole remaining component is cellulose. The diameter of fibril after bleaching decreased to 1.1 ± 0.06 µm, which is also affiliated to severe lignin removal.

### Chemical composition of *Eichhornia crassipes* fibers

The impact of pulping and bleaching pretreatments on the chemical composition of NRFs for obtaining bleached cellulose was summarized in Table 1. As represented in the table, raw NRFs consisted of cellulose (~ 33.20%), hemicellulose (~ 19.50%), and lignin (~ 7.20%). With alkaline pulping treatments, the cellulose content increased to ~ 53.20%, whereas hemicellulose and lignin got reduced to ~ 7.50% and ~ 3.85%, respectively. On further bleaching process, it was found that a further remove hemicellulose (0.72%) and lignin (1.16%) was occurred after 13 times bleaching, indicating the cleavage of ether linkages between hemicellulose and lignin in NRFs, confirming the elimination of hemicellulose and lignin achieved^26^ This outcome supported by with that gotten by TGA/DTG analysis and also agreed with other studies that published previously^27,28^

**Table 1:** Chemical composition data of the raw *Eichhornia crassipes* fibers before and after pulping and bleaching processes.

Material/treatment process	Cellulose (%)	Hemicellulose (%)	Lignin (%)
Raw NRFs	33.17 ± 0.2	19.51 ± 0.4	7.24 ± 0.1
NRFs after once pulping	49.58 ± 0.6	11.37 ± 0.3	5.53 ± 0.2
NRFs after twice pulping	53.19 ± 0.5	7.52 ± 0.1	3.84 ± 0.3
NRFs after 7 times bleaching	63.72 ± 0.8	3.84 ± 0.4	2.31 ± 0.3
NRFs after13 times bleaching	77.28 ± 0.5	0.72 ± 0.1	1.16 ± 0.2

### Atomic force microscopy (AFM)

**Figure 3:**
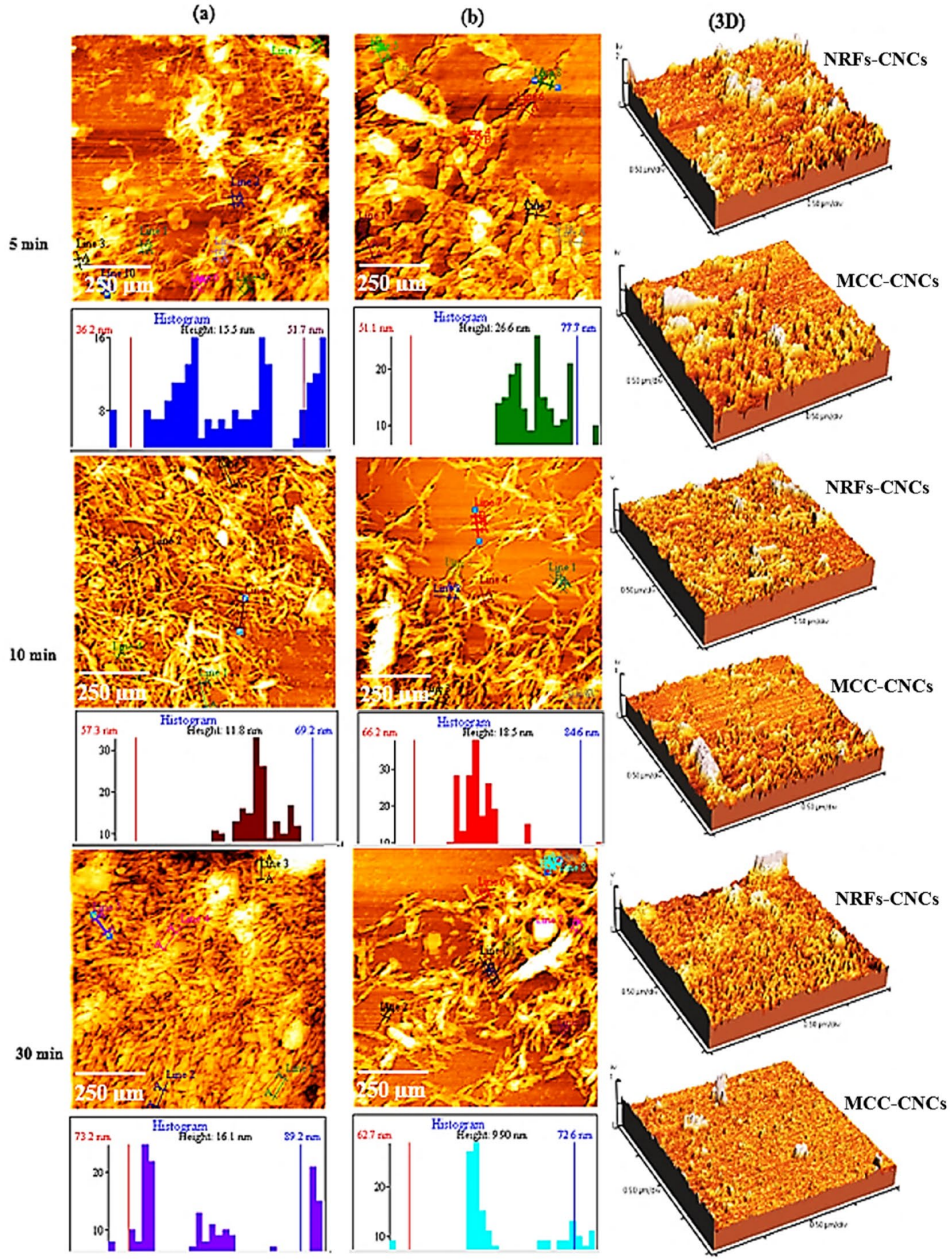
AFM topology images of CNCs extracted from NRFs (**a**) and MCC (**b**) by acid hydrolysis durations (i.e., 5, 10, and 30 min).

The morphological structures and dimension scales of extracted CNCs were studied by AFM technique, in order to point out the impact of acid hydrolysis durations (i.e., 5, 10, and 30 min) on the size of CNCs, as depicted in Fig. 3. Microcrystalline cellulose (MCC) was also provided as a reference (experimental control). It could be obviously seen from Fig. 3a that CNCs derived from NRFs appeared as fibril-like shapes with an average length of 453.50 nm and width of 15.50 ± 0.06 nm for acid hydrolysis duration of 5 min. In addition, small CNCs contents have existed of about 428.10 ± 0.12 nm in length and 51.70 ± 0.04 nm in width that may be due to the nanofibrils agglomeration. Whereas the average lengths of MCC-based CNCs were approximately 349.70 ± 0.17 nm and width of 26.6 ± 0.10 nm as illustrated in Fig. 3b. This finding was basically consistent with that reported elsewhere^29^, when they found that CNCs extracted from wood pulps were longer compared to MCC-based CNCs. With increment the acid hydrolysis time to 10 min, the average length of extracted CNCs was reduced to 351.90 ± 0.23 nm and width of 11.8 ± 0.07 nm. Also, the average length of MCC-based CNCs was reduced to 305.20 ± 0.25 nm and a distribution width of 18.50 ± 0.19 nm. Interestingly, it was observed that MCC-based CNCs have the broadest width distribution compared to CNCs derived from NRFs. The reason was probably attributed to the bundles formation from parallel-packed straight nanocrystals or packing of twisted nanocrystals^29^. On further increment of the aid hydrolysis time up to 30 min, the average length of CNCs derived from NRFs decreased to 307.60 ± 0.30 nm but its width was 16.10 ± 0.12 nm. The increase in the width for the latter was apparently due to the clusters effect which caused by capillary forces between CNCs during water vaporization^30^. On the other hand, AFM-3D images showed that CNCs topology structure and surface roughness were mostly affected by increasing the acidic hydrolysis durations and smoothness with homogeneous surfaces were noticed as shown in Table 2. This evidences that the isolate cellulose nanocrystals from *Eichhornia crassipes* were achieved and the amorphous phases broken up under the harsh acid hydrolysis time, releasing the one type of the crystallite morphologies.

**Table 2:** RMS roughness data obtained from AFM experiments.

Specimen name		Acidic hydrolysis duration		
		5 min	10 min	30 min
NRFs-CNCs	Rms rough (Rq)	12.930 ± 0.2 nm	7.217 ± 0.15 nm	6.845 ± 0.1 nm
	Ave rough (Ra)	9.421 ± 0.1 nm	5.537 ± 0.3 nm	5.217 ± 0.2 nm
MCC-CNCs	Rms rough (Rq)	11.140 ± 0.4 nm	10.430 ± 0.2 nm	3.350 ± 0.3 nm
	Ave rough (Ra)	7.470 ± 0.3 nm	7.477 ± 0.1 nm	1.889 ± 0.12 nm

### Raman spectroscopy

**Figure 4:**
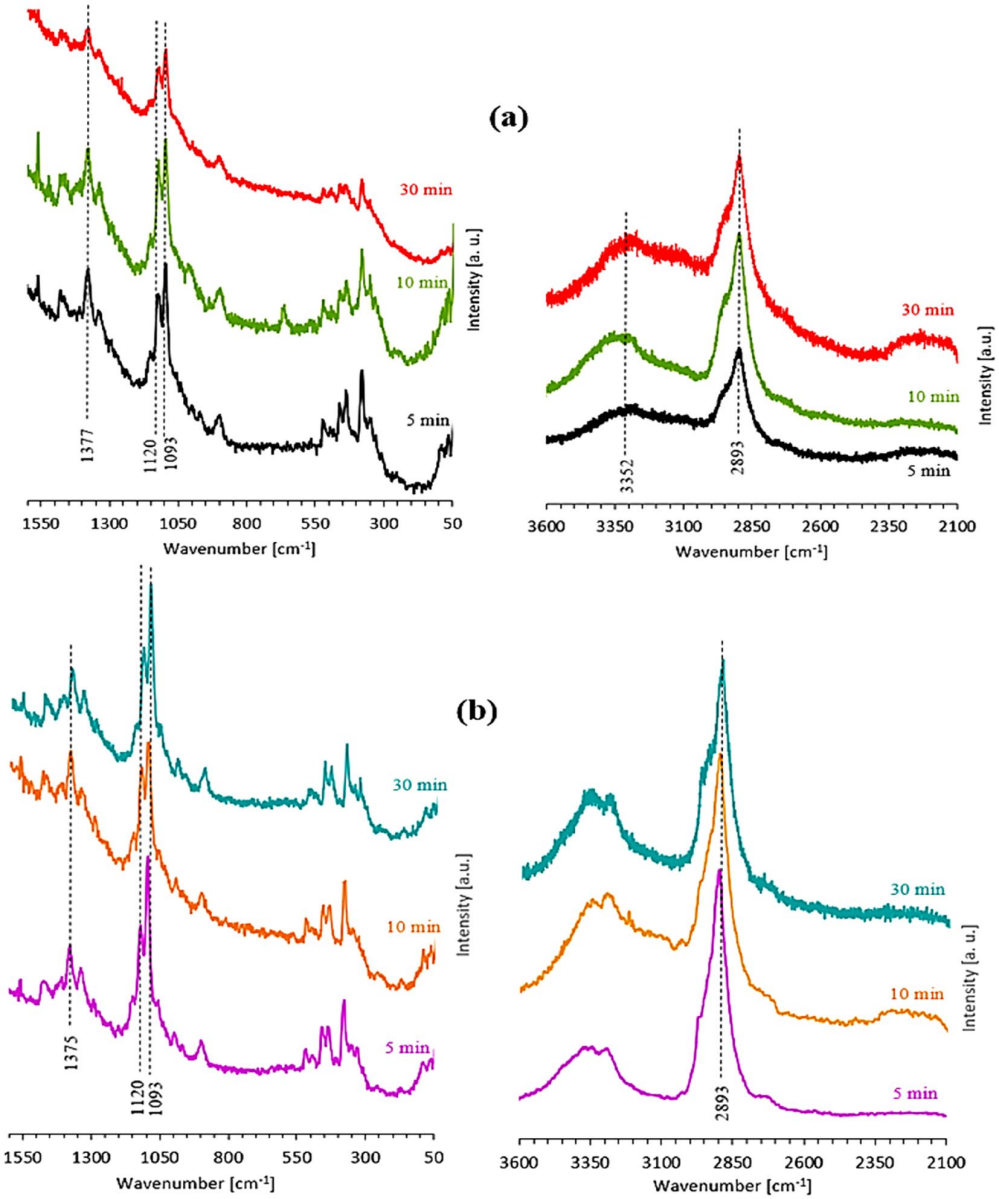
Raman spectra of CNCs derived from NRFs (**a**) and CNCs based-MCC (**b**) ranging from 50 to 3600 cm^−1^.

Raman spectra were used to identify specific peak positions of the CNCs and to detect the chemical functionalities presented in the extracted cellulose in the range of 50 to 3600 cm^-1^. For better visualization, two spectral regions were selected as given in Fig. 4. Raman investigations (showcased in Fig. 4a-b) of CNCs derived from NRFs and microcrystalline cellulose (MCC) which used for comparison. It could be obviously seen from Fig. 4a that the major peaks of CNCs for acid hydrolysis duration of 5 min were located at 1093 cm^−1^, 1120 cm^-1^, and 1377 cm^−1^ in the region of 50–1550 cm^−1^. These bands were attributed to asymmetric, symmetric β-(1,4)-glycosidic links stretching, and HCC/HCO bending, respectively^31,32^. Moreover, a massive peak at 2893 cm^−1^ which assigned to -CH_2_ stretching mode. Typical Raman peaks of CNCs have reported elsewhere^33–35^. This may be implied that the majority of extracted CNCs from NRFs was achieved. With increasing the acid hydrolysis time to 10 min, it was noticed that the intensities of these bands increased at 1093 cm^−1^, 1120 cm^−1^, and 2893 cm^−1^, indicating the high crystalline CNCs obtained. However, on further increment of the extraction time to 30 min, it was noted that their intensities got reduced especially bands at 1093 cm^−1^, 1120 cm^−1^, and 1377 cm^−1^^36^ (Fig. 4a). This reduction in the peak intensities could therefore be correlated to the fission of interchain linkages (glycosidic linkages) in CNCs and should probably drive to the amorphous form of cellulose^37,38^. In contrast, it was amazingly noted that there were no significant changes in the intensities of peaks for CNCs based-MCC samples under the acid hydrolysis durations (i.e., 5–30 min) as presented in Fig. 4b. General speaking, the particle size and the crystallinity degree of CNCs derived from agro-residues can be affected by harsh acidic hydrolysis durations and the optimum extraction time was found to be 10 min, in which duration of 10 min yielded 1.2 g nanocellulose from hydrolysis of 10 g bleached pulp.

### Thermogravimetric analysis

**Figure 5:**
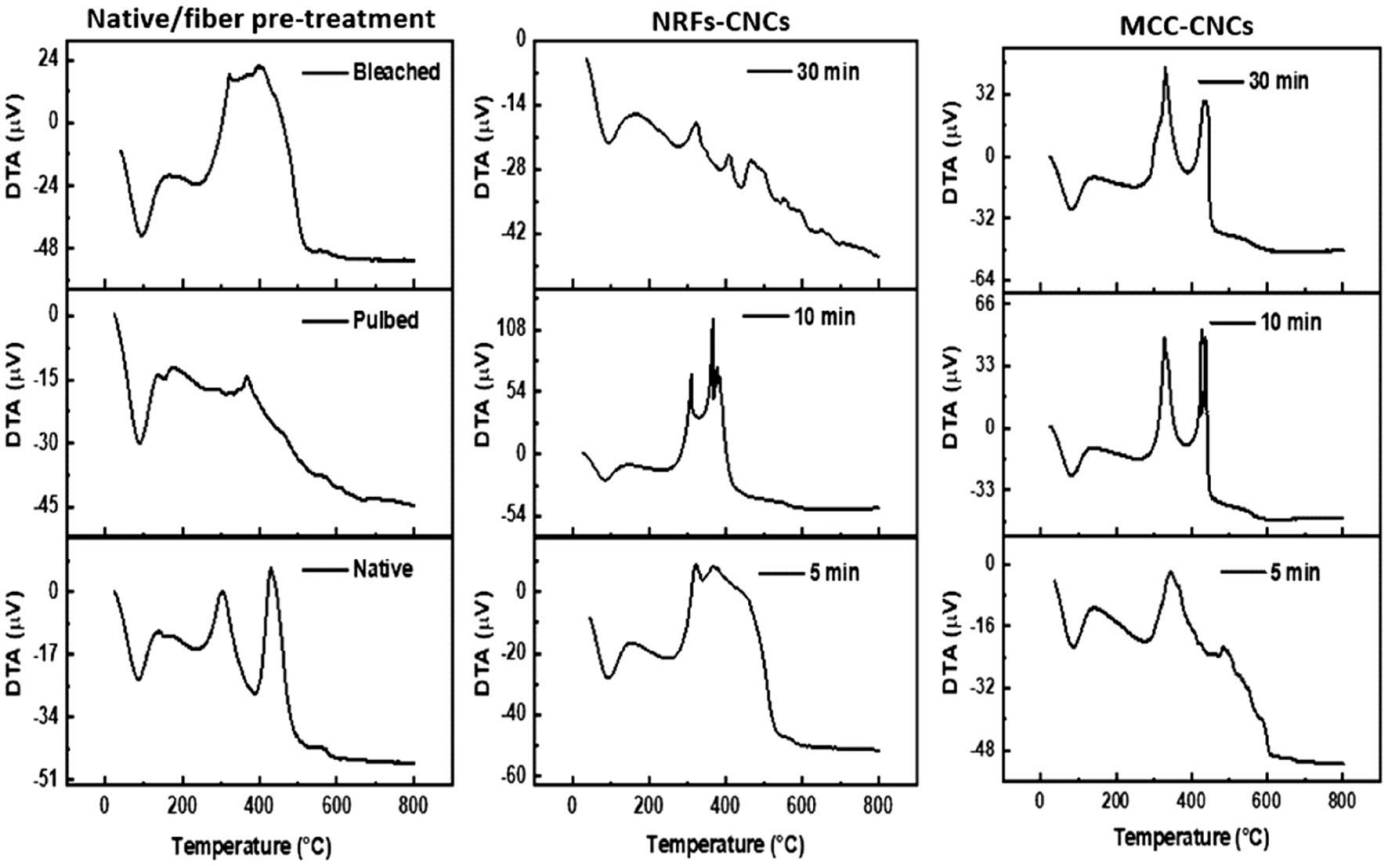
DTA thermograms of the investigated CNCs samples under air atmosphere.

TGA and DTA analysis as a helpful tool was utilized through this study to confirm the extraction of CNCs from NRFs. Moreover, the thermal stability and degradation were also affirmed from the DTA measurements. The DTA and TGA thermograms were illustrated in Fig. 5. The thermal degradation (DTA) of native fibers exhibited three peaks in the temperature range from room temperature (RT) up to 800 °C. The first endothermic peak at 80 °C was due to the evaporation of physically adsorbed water. The second exothermic peak in the temperature range 260 °C up to 340 °C was associated with the decomposition and consumption of cellulosic chain degradation^39,40^. The third exothermic peak in the temperature interval (410–460 °C) is originated from the burning of the lignin. The DTA thermogram of the pulped fibers exhibited one endothermic peak originated from the adsorbed water molecules while the other exothermic peak at 390 °C is due to the CNCs consumption. It was observed that the exothermic peak that represents the degradation of cellulosic chain was reduced to large extent. For the bleached sample, one endothermic peak represents removing the adsorbed water was observed at 93 °C.for the bleached sample, the main exothermic peak of CNCs (315–407 °C) was shifted to lower temperature compared to pulped sample. This can be explained based on the sulfate groups on the surface, that accelerates the thermal degradation of the bleached samples^41^. For the CNCs samples, the effect of hydrolysis time has a remarkable impact on the thermal stability of derived CNCs. The DTA thermograms of the 5 min, 10 min, and 30 min for bleached sample was displayed in Fig. 5, respectively. The thermal degradation (DTA) of the samples demonstrates three thermal events representing the adsorbed water molecules, sulfuric group and CNCs burning respectively.

**Figure 6:**
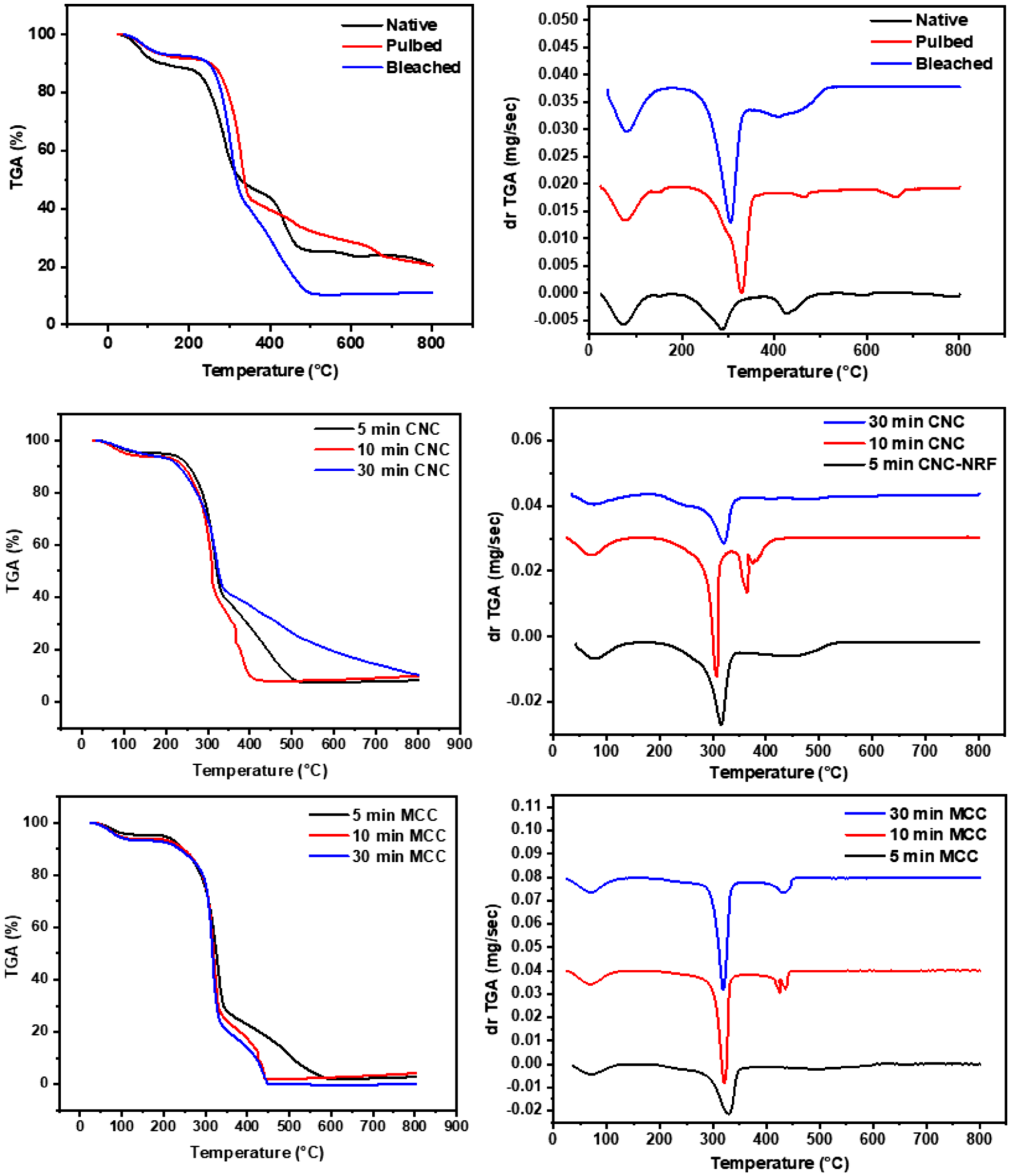
TGA/DTG curves of the investigated CNCs samples under air atmosphere.

The MCC-CNCs sample was measured for comparison. The thermal degradation of the MCC-CNCs sample was analogues to that of the bleached sample except the peak location was shifted to higher temperature. The less thermal stability of the bleached sample was attributed to the sulfur group anchored to its surface. From another point of view and as confirmed by Raman measurements, the derived CNCs from NRFs can be affected by harsh acidic hydrolysis treatment. Additional analysis represented in TGA and DTG were performed to get a deep understanding of the thermal stability beside calculating some valuable parameters such as activation energy. The native fiber sample displayed three weight losses at 102 °C, 402 °C, and 464 °C corresponding to water molecule evaporation, hemicellulose and lignin consumption, and CNCs burning out. The same three peaks of DTG curve of native sample at 72 °C, 289, and 425 °C assigned to humidity removing, hemicellulose and lignin burning, and CNCs consumption respectively. The DTG thermogram of the pulped and bleached samples confirming the removal of hemicellulose and lignin (Fig. 6). The TGA curves for the acid treated samples demonstrated two weight losses attributed to the moisture removing, and CNCs consumption, respectively. The DTG curves of the acid treated samples for different acid hydrolysis times had a single peak which assigned to the CNCs burning. The two weight losses of the TGA curves of the MCC-CNCs curves and two peaks of the DTG thermogram were due to the evaporation of the water and cellulose burning.

**Figure 7:**
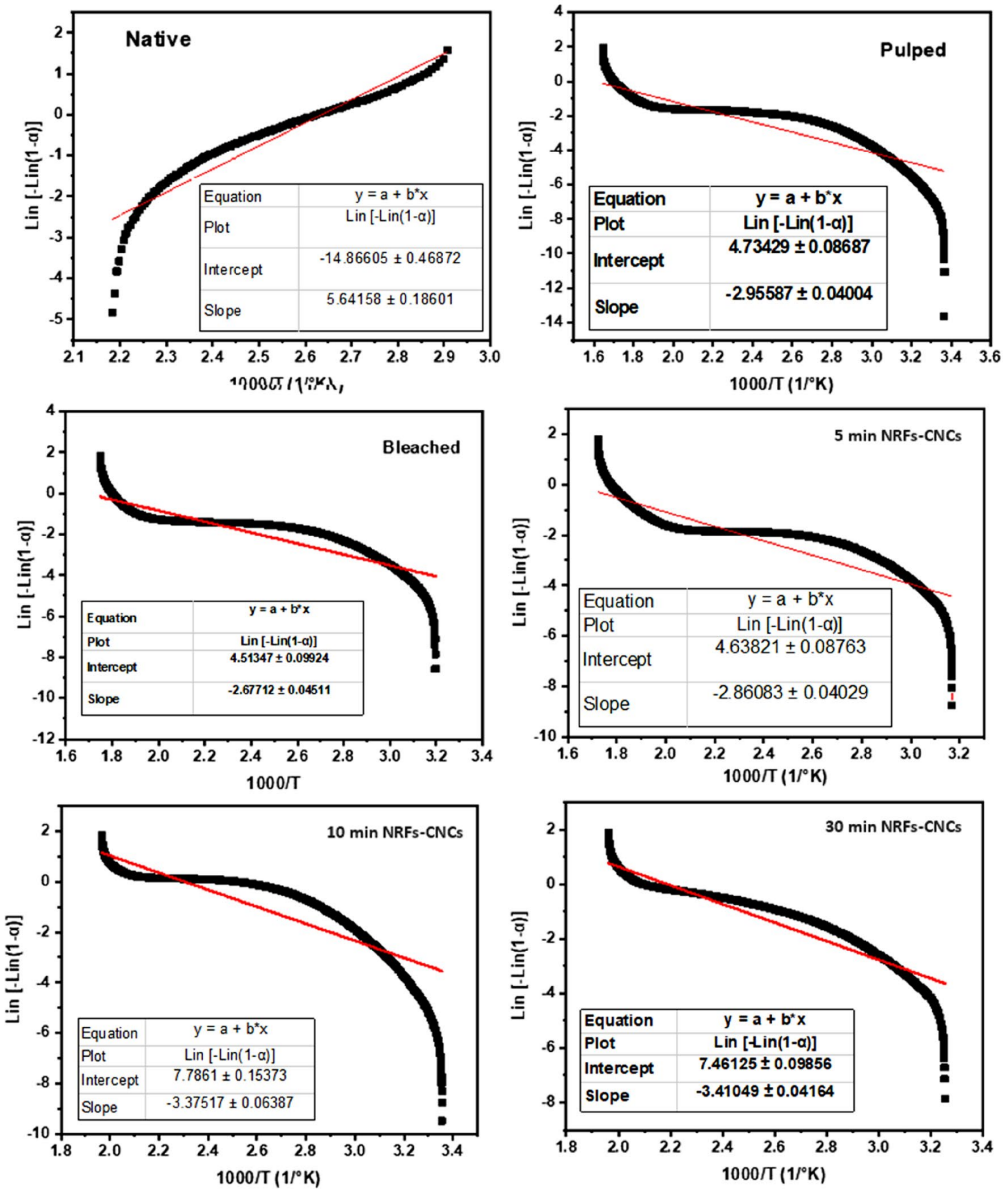
The relation between ln [ln (1-x)] and 1000/T for native, pulped, bleached, and NRFs- CNCs samples.

Activation energy can be defined as the amount of energy that the reactant requires to be converted into the product^26,27^. The two well established models for calculating activation energy are the Distributed Activation Energy Model (DAEM) and Coats Redfern method. The Coats Redfern model is based on Arrhenius equation is the most appropriate reaction model for studying the decomposition of cellulose^42^. The kinetic study has been conducted under the non-isothermal condition at a heating rate of 10 °C min^−1^ using the Coats and Redfern method. The kinetic studies of the investigated samples were obtained via a pseudo-first-order kinetic model. The Coats and Redfern model can be used to detect the kinetics parameters as expressed in the following formula^43,44^:

1 $$\ln \left[ { - \ln \left( {1 - x} \right)} \right] = \ln \frac{{ART^{2} }}{{\beta E_{a} }} - \frac{{E_{a} }}{RT}$$

where A is the pre-exponential parameter, β is the heating rate, and R is the universal gas constant (8.3143 Jmol^−1^ K^−1^). E_a_ and T are the activation energy and temperature (K) respectively. The above-mentioned equation can be visualized as straight-line equation. The activation energy can be calculated from the slope of the relation between ln [ln (1-x)] on Y-axis versus 1000/T on X-axis as shown in Fig. 7. Additional thermodynamic parameters represented in change of the entropy (ΔS^o^) , enthalpy (ΔH^o^), and Gibbs free energy (ΔG^o^) were calculated using the Eqs. 2, 3, and 4^45^:

2 $$\Delta H = E_{a} - R.T$$

3 $$\Delta S = R.\ln \left( {\frac{A.h}{{K.T}}} \right)$$

4 $$\Delta G = \Delta H - T.\Delta S$$

where h and K is Blank and Boltzmann constants respectively.

The activation energy of native fiber showed a higher value compared to other samples. The increase in the activation energy of the native sample was ascribed to the lignin decomposition. This behavior could be related to the difficulty of lignin decomposition and hence a higher temperature was required for its degradation. After modification and acid treatment of native sample, the acid treated sample for 30 min demonstrated a higher activation energy (compared to other treated samples) that related to higher decomposition temperature. This finding was in a good agreement with DGA data, where the peak of 30 min CNCs sample was shifted to higher temperature compared to other acid treated samples. The thermodynamic parameters (ΔH, ΔG, ΔS) for all samples was also presented in Table 3. The amount of heat content within the system was defined as the Enthalpy, while the Enthalpy change (ΔH) is the amount of the adsorbed or released energy during reaction at fixed pressure. The negative value of the ΔH is related to the released energy, while the positive value is due to the adsorbed energy from the surroundings to initiate the reaction. The only positive value of ΔH is attained for native fiber, demonstrating the consumption of hemicellulos and lignin. The negative value of treated samples exhibited a negative value that correlated to the exothermic reaction of crystallization. The Gibbs free energy (∆G) was defined as the amount of free energy in a system that is available. It was observed that the ∆G of native and bleached samples are approximately the same. For the acid treated samples the ∆G increases as the reaction time increases. referring to the above studied thermal parameters and comparing the Raman results, it can be concluded that the 10 min acid treated sample is the best among other prepared samples.

**Table 3:** The thermal kinetic parameters of the prepared native, pulped, bleached, and NRFs- CNCs samples.

Sample name	E_a_ (kJ/mol)	∆S (J/mol °K)	∆H (kJ/mol)	∆G (kJ/mol)
Native	46.876	0.182.08	4.0635	177.378
Pulped	24.563	0.266	− 1.939	185.436
Bleached	22.162	0.268	− 1.7176	178.487
5 min	23.773	0.267	− 1.8687	182.467
10 min	28.046	0.245	− 2.1647	210.918
30 min	28.341	0.256	− 1.9524	291.576

## Conclusions

In the current work, a high purity CNCs was successfully isolated from *Eichhornia crassipes* after a series of pulping and bleaching pretreatments, followed by acid hydrolysis at different durations ranging from 5 to 30 min. SEM and AFM analysis verified the morphological change of the NRFs before and after the chemical treatments, indicating that the alkaline and bleaching processes efficiently eliminated the hemicellulose and lignin, as indicated in chemical composition measurements. Moreover, AFM-3D images exhibited the CNCs topology, surface roughness were mostly affected by increasing the acidic hydrolysis durations. Also, Raman spectra revealed that the particle size and the crystallinity degree of NRFs-CNCs can be affected by harsh acidic hydrolysis durations and the optimum extraction time was found to be 10 min. The thermogravimetric and its derivative (TGA/DTG) analysis of the NRF-CNCs and MCC-CNCs was achieved, and the outcomes demonstrated the disappearance of the DTG peaks which assigned to the hemicellulose and lignin after chemical treatments, confirming the elimination of both components compared to the native NRFs. Referring to the above studied thermal parameters and comparing the Raman results, the 10 min acid treated sample is the best among other prepared samples. Increasing hydrolysis duration fostered thermal stability, especially for NRFs based CNCs. Results analyzed in this paper promote Nile rose as a competitive precursor for nanocellulose extraction particularly for biobased humidity nano-sensor fabrication purposes.

## Data Availability

The obtained data is provided within the manuscript.
